# Use and need for dental prostheses in oral health-related daily activities among older adults: a cross-sectional study based on SB Brasil 2023

**DOI:** 10.1590/1980-549720260022.supl.1

**Published:** 2026-07-20

**Authors:** Gustavo Silva Costa, Sara Antunes Rocha, Samuel Trezena, Fabrício Emanuel Soares de Oliveira, Hercílio Martelli, Daniella Reis Barbosa Martelli, Simone de Melo Costa, Verônica Oliveira Dias

**Affiliations:** I Universidade Estadual de Montes Claros – Montes Claros (MG), Brazil.

**Keywords:** Oral health, Aged, Quality of life, Epidemiological survey

## Abstract

**Objective:**

To assess the association between the use and need for dental prostheses and the presence of oral health impacts on daily activities, measured using the Oral Impacts on Daily Performance (OIDP) instrument, among older adults in Brazil.

**Methods:**

This cross-sectional study used data from the 2023 Brazilian National Oral Health Survey (SB Brasil), including 9,696 individuals aged 65 to 74 years, with clinical and sociodemographic information. The dependent variable was the presence of oral health impacts on daily activities, assessed by the OIDP and defined as the report of at least one negative impact. Independent variables included sociodemographic characteristics, use and need for dental prostheses, self-perceived need for prosthetic replacement, edentulism, and implant-based rehabilitation. Descriptive analyses and Poisson regression with robust variance were performed, accounting for the complex sampling design, and Prevalence Ratios (PR) and 95% Confidence Intervals (95%CI) were estimated).

**Results:**

Oral health impacts on daily activities were most frequent for eating (21.0%), smiling (16.1%), and emotional status (13.3%). Female gender (PR 1.07; 95%CI 1.01–1.14), self-perceived need to use or replace prostheses (PR 1.32; 95%CI 1.24–1.41), and need for upper prosthesis (PR 1.18; 95%CI 1.10–1.27) were associated with higher prevalence of impacts, while the use of implant-supported prostheses was a protective factor (PR 0.90; 95%CI 0.83–0.97).

**Conclusion:**

Prosthetic status is central to the relationship between oral health and quality of life in older adults, reinforcing the need for public policies that expand access and equity in prosthetic rehabilitation.

## INTRODUCTION

Population aging has altered morbidity patterns and posed challenges to the organization of health services due to the increased demand for care^1–3^. In Brazil, this process has expanded discussions surrounding the health agenda, with implications for multiple areas of care, including oral health care for older adults^2,4^.

In this context, edentulism is associated with adverse functional, nutritional, and psychosocial outcomes. Oral rehabilitation with dental prostheses is widely used to restore masticatory function and aesthetics; however, its effects may vary according to the type of prosthesis, health conditions, individual expectations, and access to dental services. Evidence indicates an association between prosthetic rehabilitation and improvements in Oral Health-Related Quality of Life (OHRQoL), particularly in the functional and psychological domains, reinforcing the need for approaches that consider broader dimensions of oral health care in the aging population^5–10^.

The historical evolution of dental prosthesis use among older Brazilian adults, as well as evidence regarding the need for prosthetic rehabilitation, can be understood based on data from the National Oral Health Survey (*Pesquisa Nacional de Saúde Bucal –* SB Brasil). Between 2003 and 2010, an increase was observed in the use of complete upper (57.91 to 63.1%) and lower (24.84 to 37.5%) dentures. This multifactorial phenomenon is related both to the persistence of tooth loss, assessed through the "missing" component of the DMFT index, and to the expansion of the availability of and access to prosthetic rehabilitation services. Although a progressive reduction in edentulism has been reported, the high proportion of missing teeth maintains removable prosthetic rehabilitation as the primary strategy for functional restoration within public health services, as alternatives such as fixed prostheses and implant-supported rehabilitations remain limited in the Brazilian Unified Health System (*Sistema Único de Saúde* – SUS), thereby restricting their population-level reach^9–12^.

In parallel, a slight increase in the use of single-unit fixed restorations was observed. In 2003, the use of fixed bridges was 0.79% in the lower arch and 1.87% in the upper arch, increasing to 3.8% in 2010. Although still uncommon, these findings suggest gradual changes in the pattern of tooth loss and in access to more conservative treatments^11–13^, reinforcing the need to investigate the association between prosthetic condition and OHRQoL. Such investigations require the use of specific and validated instruments, such as the Oral Impacts on Daily Performance (OIDP) index.

The OIDP assesses the impact of oral health conditions on daily activities, encompassing physical, psychological, and social dimensions. In Brazil, the validated version of the instrument demonstrated adequate psychometric properties among adults aged 50 to 74 years, supporting its applicability in this population^14^. Recent evidence^15^ also suggests that the association between prosthetic rehabilitation and OHRQoL is not uniform. Although the presence of prostheses represents an important component in this assessment, its effects vary according to the pattern of tooth loss, such as anterior tooth loss or posterior extension. Consequently, certain types of prostheses may not be associated with significant improvements in this outcome.

Despite these advances, important gaps remain in the literature, primarily due to the predominance of local studies or investigations involving broad age ranges, which limits analyses based on representative population surveys. Furthermore, studies comprehensively assessing the use and need for dental prostheses, as well as their functional, psychological, and social impacts within the context of aging and inequalities in access to dental services, remain scarce. Consequently, national epidemiological evidence relating prosthetic status to the impact of oral health on the daily activities of older adults is still limited, highlighting the need for nationwide studies^16,17^.

Thus, the present study aimed to assess the association between the use of and need for dental prostheses and the impact of oral health on the daily activities of older adults in Brazil.

## METHODS

### Study design and population

This cross-sectional study used secondary data from SB Brasil 2023, which employed a stratified cluster sampling design to ensure national representativeness, with coverage across the five macroregions, Brazilian states, and state capitals^7^. A total of 9,745 older adults aged 65 to 74 years were evaluated. Individuals with incomplete clinical data were excluded, resulting in a final sample of 9,696 participants.

### Data collection and study variables

Data were collected through household interviews and standardized clinical examinations conducted by calibrated examiners, following the World Health Organization (WHO) methodology for oral health surveys. The questionnaire gathered sociodemographic information (gender, skin color/race, educational level, and family income) as well as information related to service utilization (such as the use of dental services and self-perception regarding the use of and need for dental prostheses).

The dependent variable was OHRQoL, assessed using the OIDP, which comprises three domains: the physical domain, related to difficulties in performing activities such as chewing or speaking; the psychological domain, encompassing impacts on emotional well-being, self-esteem, and self-perception; and the social domain, referring to limitations in social interactions and participation^18,19^. According to previously established methodology^20^, participants who reported at least one negative impact were classified as having an impact, whereas those who responded negatively to all items were classified as having no impact. The OIDP assesses difficulties or impairments in the performance of daily activities attributed to oral problems within a defined reference period, constituting a synthetic, validated, and widely used measure in population-based epidemiological studies^21^.

The independent variables were selected based on previous evidence from SB Brasil and their theoretical relevance to the analysis of OHRQoL. Sociodemographic variables included gender (female; male), self-reported skin color/race (White; Black; Brown; Yellow; Indigenous), according to the classification adopted by the Brazilian Institute of Geography and Statistics (*Instituto Brasileiro de Geografia e Estatística* – IBGE), educational level (no formal education; up to 8 years of schooling; more than 8 years of schooling), and monthly family income (<1 minimum wage; 1–2 minimum wages; >2 minimum wages). Region of residence (North; Northeast; Central-West; Southeast; South) and variables related to dental service utilization were also considered, including use of dental services within the previous year (yes; no) and type of service used (public; private; health insurance plan)^7^.

Regarding clinical and rehabilitation conditions, the following variables were analyzed: edentulism (yes; no), use of dental prostheses (yes; no), need for upper and lower prostheses (yes; no), self-perceived need to use or replace prostheses (yes; no), and presence of implant-supported prostheses or dental implants (yes; no)^7^. All variables were categorized according to the SB Brasil criteria, ensuring comparability with previous national studies.

Participants with missing clinical data related to the use of and need for dental prostheses or edentulism (n=25) were excluded from the sample. Responses classified as "do not know" or refusals to answer were treated as missing data in the analyses.

### Statistical analysis

Statistical analyses were performed using the Statistical Package for the Social Sciences (SPSS), version 27.0 (IBM Corp., Armonk, NY, USA). Initially, descriptive analyses were conducted by calculating absolute and relative frequencies. Associations between OHRQoL and the independent variables were assessed using Poisson regression with robust variance estimation, taking into account the complex sampling design of SB Brasil (sampling weights, strata, and primary sampling units).

In the bivariate analysis, crude prevalence ratios (PRs) and their respective 95% confidence intervals (95%CIs) were estimated. Variables with p<0.20 were included in the multivariable model, whereas those with p<0.05 were retained in the final model.

### Ethical considerations

The SB Brasil project followed the principles of the Declaration of Helsinki and was approved by the Brazilian National Research Ethics Committee (approval No. 4.823.054). All participants provided informed consent prior to participation. As the present study used publicly available and anonymized secondary data, no additional ethical approval was required.

#### Data availability statement:

The entire dataset supporting the results of this study is available upon request from the Ministry of Health.

## RESULTS

The distribution of participants across the Brazilian macroregions showed a higher concentration in the Northeast (33.6%; 95%CI 32.6–34.5) and the lowest concentration in the Central-West region (13.8%; 95%CI 13.1–14.5). Most participants were female (61.9%; 95%CI 60.9–62.8) and self-identified as Brown (46.5%; 95%CI 45.5–47.5). The mean number of years of formal education was 6.96 (±4.57), and only 8.2% (95%CI 7.6–8.7) of the sample reported having completed higher education. Regarding income, most participants reported earning between 1 and 2 minimum wages (35.6%; 95%CI 34.5–36.7). Smaller proportions reported monthly incomes of more than 5 to 10 minimum wages (6.2%; 95%CI 5.6–6.7) and more than 10 minimum wages (2.1%; 95%CI 1.8–2.4) (Table 1).

**Table 1:** Sociodemographic characteristics of Brazilian older adults, SB Brasil 2023.

Characteristic		n	%	95%CI
Region				
	North	2,231	23.0	22.1–23.8
	Northeast	3,258	33.6	32.6–34.5
	Central-West	1,344	13.9	13.1–14.5
	Southeast	1,451	15.0	14.2–15.6
	South	1,412	14.6	13.8–15.2
Gender				
	Male	3,693	38.1	37.1–39.0
	Female	6,003	61.9	60.9–62.8
Self-reported race/skin color				
	White	3,578	37.5	36.4–38.4
	Black	1,350	14.1	13.4–14.8
	Yellow	129	1.4	1.35–1.60
	Brown	4,447	46.5	45.5–47.5
	Indigenous	50	0.5	0.4–0.69
Family income (minimum wages)				
	Up to one	1,987	28.3	27.2–29.3
	1 to 2	2,501	35.6	34.5–36.7
	More than 2	2,531	36.1	34.9–37.1
Years of schooling				
	No formal education	1,233	13.0	12.3–13.7
	Up to 8	4,573	48.2	47.2–49.2
	More than 8	3,675	38.8	37.7–39.7

CI: Confidence interval.

In recent years, 56.2% (95%CI: 55.2–57.1) of participants reported not having sought dental care. Among the 4,206 older adults who had used dental services (43.8%; 95%CI 42.8–44.8), 47.9% (95%CI 46.4–49.4) used private services, 45.4% (95%CI 43.9–46.9) sought care through public services, and 6.6% (95%CI 5.9–7.4) used health insurance plans. Less than 1% (0.6%; 95%CI 0.4–0.8) reported using other types of services. Slightly more than half of the participants (53.2%; 95%CI 52.1–54.1) reported needing to use or replace a complete denture, whereas 19.3% (95%CI 18.5–20.1) reported having teeth or prostheses supported by implants.

**Figure 1:**
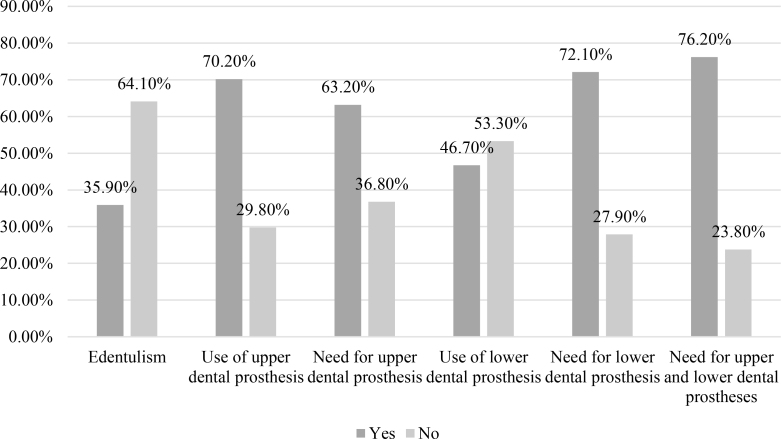
Edentulism, use of dental prostheses, and need for dental prostheses among Brazilian older adults, SB Brasil, 2023.

Figure 1 presents the prevalence of edentulism and the use of and need for dental prostheses among older Brazilian adults. Overall, 35.9% (95%CI 34.9–36.8) of participants were edentulous. The use of upper dental prostheses was reported by 70.2% (95%CI 69.3–71.1) of participants, whereas 46.7% (95%CI 45.7–47.6) reported using lower dental prostheses. The need for upper and lower prostheses was identified in 63.2% (95%CI 62.1–64.1) and 72.1% (95%CI 71.1–72.9) of participants, respectively. Regarding the overall need for prosthetic rehabilitation in both arches, 23.8% (95%CI 22.9–24.6) of older adults did not require prostheses, 22.6% (95%CI 21.7–23.4) required partial prostheses in both arches, and 23% (95%CI 22.1–23.8) required complete prostheses in both the upper and lower arches.

Regarding the types of prostheses used, among participants who reported using upper dental prostheses, nearly half (49.8%; 95%CI 48.8–50.8) used removable complete dentures; 13.6% (95%CI 12.9–14.2) used removable partial dentures; and 3% (95%CI 2.6–3.3) used one or more fixed bridges. Additionally, 2.6% (95%CI 2.3–2.9) used fixed complete dentures (overdentures), and 1.2% (95%CI 0.9–1.4) used a combination of fixed and removable prostheses. For the lower arch, 26.2% (95%CI 25.3–27.0) used removable complete dentures; 14.7% (95%CI 14.0–15.4) used removable partial dentures; and 1.9% (95%CI 1.6–2.2) had one or more fixed bridges. A small proportion reported using fixed complete dentures (2.6%; 95%CI 2.3–2.9) or a combination of fixed and removable prostheses (1.3%; 95%CI–1.5). Regarding prosthetic needs, 36.9% (95%CI 35.9–37.8) of participants required a complete upper denture, whereas 18.2% (95%CI 17.4–19.0) required a prosthesis to replace more than one missing tooth, and 5.1% (95%CI 4.7–5.6) required a combination of fixed and removable prostheses. In the lower arch, 27.3% (95%CI 26.3–28.1) required a complete denture, 32.6% (95%CI 31.6–33.5) required replacement of more than one tooth, and 9.3% (95%CI 8.7–9.8) required a combination of prosthetic types.

**Figure 2:**
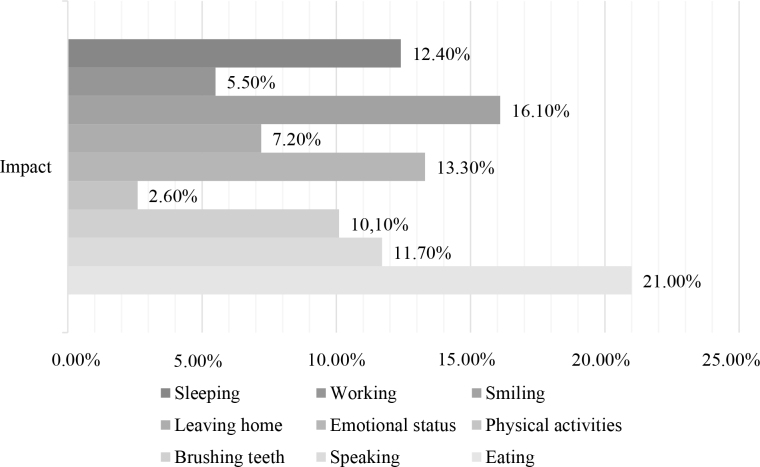
Oral impacts on daily performance among Brazilian older adults, SB Brasil, 2023.

More than half of the older adults (56.1%; 95%CI 55.1–57.0) reported experiencing at least one impact on daily activities related to oral health, whereas 2.3% reported impacts across all assessed dimensions. Eating (21.0%) and smiling (16.1%) were the most frequently affected activities. Impacts on emotional well-being were reported by 13.3% of participants (Figure 2). Associations between oral health impacts and the independent variables are presented in Table 2.

**Table 2:** Association between oral health impacts on quality of life and sociodemographic, clinical, and prosthesis use and need characteristics among Brazilian older adults, SB Brasil, 2023.

Characteristic		Without impact	With impact	PR	95%CI	p-value*
		n (%)				
Region of Brazil						
	Central-West	489 (36.6)	848 (63.4)	1.08	1.02–1.14	**0.005**
	South	657 (46.6)	752 (53.4)	0.90	0.85–0.96	**0.002**
	Southeast	713 (49.5)	727 (50.5)	0.86	0.80–0.91	**<0.001**
	Northeast	1,459 (45.0)	1,782 (55.0)	0.93	0.89–0.98	**0.006**
	North	916 (41.3)	1,303 (58.7)	1		
Gender						
	Female	2,562 (42.9)	3,414 (57.1)	1.04	1.01–1.08	**0.010**
	Male	1,672 (45.6)	1,998 (54.4)	1		
Self-reported skin color						
	Indigenous	18 (36.0)	32 (64.0)	1.19	0.96–1.47	0.098
	Brown	1,928 (43.6)	2,495 (56.4)	1.05	1.01–1.09	**0.013**
	Yellow	56 (43.8)	72 (56.2)	1.04	0.89–1.22	0.545
	Black	533 (39.6)	814 (60.4)	1.12	1.06–1.18	**<0.001**
	White	1,653 (46.4)	1,910 (53.6)	1		
Education (years)						
	No formal education	482 (39.3)	745 (60.7)	1.13	1.07–1.19	**<0.001**
	Up to 8	1,959 (43.0)	2,601 (57.0)	1.06	1.02–1.10	**0.001**
	More than 8	1,704 (46.5)	1,960 (53.5)	1		
Family income (minimum wages)						
	<1	838 (42.3)	1,141 (57.7)	1.08	1.03–1.14	**0.002**
	1–2	1,012 (40.6)	1,481 (59.4)	1.11	1.06–1.17	**<0.001**
	>2	1,183 (46.9)	1,340 (53.1)	1		
Sought dental care (last year)						
	Yes	1,757 (41.9)	2,440 (58.1)	1.06	1.02–1.10	**<0.001**
	No	2,435 (45.4)	2,934 (54.6)	1		
Type of dental service						
	Public	707 (37.4)	1,183 (62.6)	1.35	1.18–1.54	**<0.001**
	Private	889 (44.7)	1,101 (55.3)	1.19	1.04–1.36	**0.009**
	Health insurance/Health plan	147 (53.6)	127 (46.4)	1		
Self-perception regarding use or replacement of dental prosthesis						
	Yes	1,706 (34.1)	3,295 (65.9)	1.46	1.40–1.51	**<0.001**
	No	2,420 (54.9)	1,986 (45.1)	1		
Teeth/prostheses with implants						
	Yes	855 (46.2)	997 (53.8)	0.94	0.90–0.99	**0.029**
	No	3,346 (43.3)	4,381 (56.7)	1		
Edentulism						
	No	2,620 (42.3)	3,567 (57.7)	1.08	1.04–1.12	**<0.001**
	Yes	1,614 (46.7)	1,845 (53.3)	1		
Use of upper prosthesis						
	No	1,257 (43.8)	1,611 (56.2)	1.02	0.96–1.04	0.933
	Yes	2,977 (43.9)	3,801 (56.1)	1		
Need for upper prosthesis						
	No	1,890 (53.1)	1,669 (46.9)	0.76	0.73–0.79	**<0.001**
	Yes	2,344 (38.5)	3,743 (61.5)	1		
Use of lower prosthesis						
	No	2,128 (41.4)	3,006 (58.6)	1.09	1.05–1.13	**<0.001**
	Yes	2,106 (46.7)	2,406 (53.3)	1		
Need for lower prosthesis						
	No	1,509 (55.9)	1,190 (44.1)	0.72	0.69–0.76	**<0.001**
	Yes	2,725 (39.2)	4,222 (60.8)	1		
Need for upper and lower prosthesis						
	Yes	2,917 (39.7)	4,430 (60.3)	1.41	1.34–1.48	**<0.001**
	No	1,317 (57.3)	982 (42.7)	1		

PR: Prevalence Ratios; CI: confidence interval.

* Statistically significant association (p<0.05).

There was a statistically significant association for the values highlighted in bold.

In the bivariate analysis (Table 2), a higher prevalence of oral health impacts was observed among participants from the Central-West region (p=0.005), compared with those from the North region (reference category); among women (p=0.010); Black (p<0.001) and Brown individuals (p=0.013); participants with lower educational attainment (p<0.001) and lower family income (p=0.002); and those who used public dental services (p<0.001). Older adults who perceived the need to use or replace complete dentures (p<0.001), were edentulous (p<0.001), or required upper (p<0.001) or lower (p<0.001) dental prostheses also reported a higher prevalence of oral health impacts. In contrast, individuals with implant-supported teeth or prostheses showed a lower prevalence of impacts (p=0.029).

In the Poisson regression model (Table 3), female gender remained significantly associated with a higher prevalence of oral health impacts on daily activities (PR 1.07; 95%CI 1.01–1.14; p=0.022). Self-perceived need to use or replace a complete denture (PR 1.32; 95%CI 1.24–1.41; p<0.001) and the need for an upper dental prosthesis (PR 1.18; 95%CI 1.10–1.27; p<0.001) were also associated with a higher prevalence of these impacts. Conversely, the presence of implant-supported teeth or prostheses was associated with a lower prevalence of oral health impacts on daily activities (PR 0.90; 95%CI 0.83–0.97; p=0.006).

**Table 3:** Poisson regression model with robust estimator between oral impacts on daily performance and sociodemographic, clinical, and prosthesis use and need characteristics among Brazilian older adults, SB Brasil, 2023.

Characteristic		*B*	PR	95%CI	p-value
Gender					
	Female	0.073	1.07	1.01–1.14	**0.022**
	Male			*Ref.*	
Self-perception regarding use or replacement of dental prosthesis					
	Yes	0.284	1.32	1.24–1.41	**<0.001**
	No			*Ref.*	
Teeth/prostheses with implants					
	Yes	-0.104	0.90	0.83–0.97	**0.006**
	No			*Ref.*	
Need for upper prosthesis					
	Yes	0.172	1.18	1.10–1.27	**<0.001**
	No			*Ref.*	

PR: Prevalence Ratios; CI: confidence interval.

*Ref.:* reference category of the analysis.

There was a statistically significant association for the values highlighted in bold.

## DISCUSSION

The findings of this study indicate that oral health continues to exert a substantial impact on the daily activities of older Brazilian adults. More than half of the participants reported at least one oral health impact, with eating, smiling, and emotional aspects representing the most affected domains. A higher prevalence of impacts was observed among women, individuals with a self-perceived need to use or replace dentures, and those requiring an upper dental prosthesis, whereas the presence of implant-supported teeth or prostheses was identified as a protective factor. In addition to objective clinical conditions, subjective and sociodemographic factors appear to play a central role in determining the impact of oral health on daily life, reinforcing the complexity of the relationship between tooth loss, prosthetic rehabilitation, and OHRQoL among older Brazilian adults.

Although not the central focus of the present study, edentulism remains an important marker of the oral health conditions of older Brazilian adults and provides context for understanding the high need for prosthetic rehabilitation observed. Previous SB Brasil studies^13,22–24^ have documented a progressive decline in edentulism rates over recent decades; however, the magnitude still observed reflects a historical pattern of mutilating dental practices and limited access to preventive actions and conservative treatments^25^. The predominance of removable complete dentures, consistently described in the national literature, particularly in SB Brasil surveys and studies evaluating prosthesis provision within SUS, reinforces that conventional prosthetic rehabilitation remains the primary strategy for addressing tooth loss among older adults within the Brazilian public health system^6,13^.

A high prevalence of need for upper and lower dental prostheses was observed, corroborating findings from previous investigations conducted in Brazil^26,27^, particularly in contexts characterized by inequalities in access to dental services. Despite the expansion of public oral health policies, the public provision of prostheses remains insufficient to meet existing demand. Moreover, much of the tooth loss occurred during adulthood, reflecting historically mutilating dental practices and a predominantly curative model of care. Therefore, the high need for prosthetic rehabilitation identified in SB Brasil 2023 reflects not only current demands on the health system but also the cumulative effect of inequities and gaps in oral health care throughout the life course.

The findings of the present study also suggest changes in comparison with previous editions of SB Brasil. Although edentulism has declined, the need for prosthetic rehabilitation has remained high, particularly regarding the growing demand for complete upper dentures. This finding may be partially explained by the self-reported nature of this information, as the absence, inadequacy, or wear of upper dentures tends to generate a greater perception of aesthetic, functional, and psychosocial impairments, including difficulties with eating, speaking, smiling, and social interaction^28–30^. Therefore, the greater demand for upper dentures may reflect not only objective clinical conditions but also an increased perception of the impact of oral health on daily activities, as assessed by the OIDP^31,32^.

The discrepancy between the use of and need for prostheses, particularly in the lower arch, may be explained by socioeconomic barriers and limitations in access to dental services. The greater need for prosthetic rehabilitation among individuals with lower income and educational attainment reflects financial constraints and restricted access to specialized dental services within the public sector. National and international evidence^22,24,33–36^ consistently demonstrates an association between unfavorable socioeconomic conditions and poorer oral health outcomes among older adults. This scenario is further aggravated by the high unmet demand for specialized care within the public health system, resulting in prolonged waiting times and delayed rehabilitative treatment^37^.

In addition to access to care, the adequacy of the prostheses used represents a central component in understanding the discrepancy between prosthetic need and use. Clinical evidence demonstrates that a considerable proportion of prostheses used by older adults present deficiencies in retention, stability, and functional adaptation, thereby compromising masticatory function, comfort, and treatment satisfaction. These limitations increase the likelihood of prosthesis replacement or discontinuation of use^30,32^. This scenario contributes to the persistence of negative impacts on OHRQoL and to the widening of inequalities in oral health care.

Self-perception of oral health was strongly associated with the perceived need for the use or replacement of prostheses and with a higher prevalence of impacts on daily activities. National evidence indicates that a positive self-perception may be related to the perceived absence of need for treatment, even in the presence of unfavorable clinical conditions^38^, suggesting adaptation to the limitations associated with aging^39^. Conversely, studies have shown that negative self-perception is associated with greater functional and psychosocial impacts, a higher number of complaints, and lower utilization of dental services^30,40^, highlighting the relevance of subjective factors in the experience of oral health.

The relationship between prosthesis use and need and quality of life, as measured by the OIDP, is evidenced by the finding that more than half of the older adults reported at least one oral health impact on their daily activities. Thes findings are consistent with the international literature^41–43^, which demonstrates a persistent association between tooth loss, need for prosthetic rehabilitation, and poorer OHRQoL. In the Brazilian context, the greater frequency of impacts related to eating, smiling, and emotional well-being reinforces that the demand for prosthetic rehabilitation extends beyond technical aspects, encompassing self-esteem, social interactions, and psychological well-being^44^.

Female gender was also associated with a greater likelihood of reporting oral health impacts on daily activities, a finding consistent with the literature demonstrating gender differences in the perception of social and psychological impacts related to oral health^45,46^. The persistence of female gender as a significant determinant of OIDP scores highlights the need for gender-sensitive oral health care strategies aimed at reducing these inequalities.

Structural factors influencing the provision and use of dental prostheses also warrant attention. The findings indicate that a substantial proportion of dental care was provided through both private and public services, demonstrating that the public health system continues to account for a considerable share of the demand for prosthetic rehabilitation. This scenario, combined with a less favorable socioeconomic profile, points to the persistence of inequalities in the provision of and access to dental services. A Brazilian study demonstrated that regions with a lower Human Development Index (HDI) and lower density of dental infrastructure exhibit greater prosthetic needs and lower rates of prosthesis use^30^, reinforcing the urgency of policies aimed at expanding prosthetic rehabilitation within SUS, with an emphasis on equity.

Within the context of SUS, prosthetic rehabilitation should be provided predominantly through Primary Health Care (PHC), given its capillarity, accessibility, and longitudinal continuity of care^47^. However, previous studies^48–50^ have shown that only a small proportion of oral health teams within PHC actually provide dental prosthesis services, resulting in the concentration of such services within specialized care settings or the private sector.

Regarding the type of rehabilitation, removable complete dentures, particularly when poorly fitted, have been associated with a greater negative impact on quality of life^51^. National studies have also demonstrated an association between the use of lower partial dentures and a higher likelihood of impacts on OIDP scores^32,52^, reinforcing that expanding service provision alone is insufficient; ensuring the quality, adequacy, and continuous monitoring of prostheses is equally essential^30^. Thus, the high prevalence of removable complete dentures and the substantial proportion of unmet prosthetic needs suggest that merely providing prostheses is not sufficient. It is also necessary to ensure their adequacy, maintenance, and comprehensive rehabilitation in order to minimize the impact on quality of life.

The use of implant-supported prostheses demonstrated a protective effect on the quality of life of older adults, corroborating studies reporting significant improvements following the placement of overdentures^53,54^. These rehabilitative approaches provide greater stability and retention compared with conventional removable prostheses, thereby favoring improved mastication, comfort, speech, and satisfaction with appearance^29,30,55^. However, the low prevalence of this type of rehabilitation reflects not only socioeconomic barriers and limitations in access to care, but also structural constraints within the Brazilian health system. Although SUS has financed these procedures since 2010, their implementation remains limited^56,57^, reinforcing the need for policies aimed at expanding access to these technologies^58^.

This study has limitations inherent to its cross-sectional design, which precludes causal inferences. In addition, the assessment of quality of life was based on self-reported information and is therefore subject to memory bias and subjectivity. Nevertheless, the national representativeness of the sample, the methodological rigor of the SB Brasil survey, and the use of validated instruments strengthen the robustness of the findings. Furthermore, the multivariable analysis allowed for the control of potential confounding factors.

In line with the literature^59^, the assessment of oral health impacts through the OIDP constitutes a strategic tool for identifying vulnerable groups and monitoring public policies. Thus, the findings reinforce that prosthetic conditions play a central role in the relationship between oral health and quality of life among older adults in Brazil, highlighting the need for policies aimed at expanding equitable, qualified, and continuous access to prosthetic rehabilitation within SUS, while addressing regional, socioeconomic, and gender inequalities.
