# Supplementary material for: Use and need for dental prostheses in oral health-related daily activities among older adults: a cross-sectional study based on SB Brasil 2023
Source: Rev Bras Epidemiol. 2026 Jul 20;29(Suppl 1):e260022supl1. doi: 10.1590/1980-549720260022.supl.1 (PMC13403697; doi:10.1590/1980-549720260022.supl.1)
Supplement: Translation

# Translation: Uso e necessidade de próteses dentárias nas atividades diárias relacionadas à saúde bucal em pessoas idosas: estudo transversal derivado do SB Brasil 2023

**DOI:** 10.1590/1980-549720260022.supl.1.2

**Authors:** Gustavo Silva Costa, Sara Antunes Rocha, Samuel Trezena, Fabrício Emanuel Soares de Oliveira, Hercílio Martelli, Daniella Reis Barbosa Martelli, Simone de Melo Costa, Verônica Oliveira Dias

**Affiliations:** I Universidade Estadual de Montes Claros – Montes Claros (MG), Brasil.

**Keywords:** Saúde bucal, Pessoa idosa, Qualidade de vida, Levantamento epidemiológico

## Abstract

**Objetivo:**

Avaliar a associação entre uso e necessidade de próteses dentárias e a presença de impactos da saúde bucal nas atividades diárias, mensurados pelo instrumento Impactos Bucais no Desempenho Diário (OIDP), em pessoas idosas no Brasil.

**Métodos:**

Estudo transversal com dados da Pesquisa Nacional de Saúde Bucal (SB Brasil) 2023, incluindo 9.696 indivíduos (65 a 74 anos), com informações clínicas e sociodemográficas. A variável dependente foi a presença de impactos da saúde bucal nas atividades diárias, avaliada pelo OIDP e definida como o relato de pelo menos um impacto negativo. As variáveis independentes incluíram características sociodemográficas, uso e necessidade de próteses dentárias, autopercepção da necessidade de substituição protética, edentulismo e reabilitação com implantes. Realizaram-se análises descritivas e regressão de Poisson com variância robusta, considerando o desenho amostral complexo, estimando-se Razões de Prevalência (RP) e Intervalos de Confiança de 95% (IC95%).

**Resultados:**

Os impactos da saúde bucal nas atividades diárias foram mais frequentes na alimentação (21%), no sorriso (16,1%) e no estado emocional (13,3%). O sexo feminino (RP 1,07; IC95% 1,01–1,14), a autopercepção de necessidade de usar ou substituir prótese (RP 1,32; IC95% 1,24–1,41) e a necessidade de prótese superior (RP 1,18; IC95% 1,10–1,27) associaram-se a maior prevalência de impactos, enquanto o uso de próteses apoiadas em implantes foi fator protetor (RP 0,90; IC95% 0,83–0,97).

**Conclusão:**

A condição protética é central na relação entre saúde bucal e qualidade de vida de pessoas idosas, reforçando a necessidade de políticas públicas que ampliem acesso e equidade na reabilitação protética.

## INTRODUÇÃO

O envelhecimento populacional tem alterado os padrões de morbidade e imposto desafios à organização dos serviços de saúde, em razão do aumento da demanda assistencial^1–3^. No Brasil, esse processo ampliou o debate na agenda sanitária, com implicações para diferentes áreas do cuidado, incluindo a atenção odontológica às pessoas idosas^2,4^.

Nesse contexto, o edentulismo associa-se a desfechos negativos funcionais, nutricionais e psicossociais. A reabilitação oral com próteses dentárias é amplamente utilizada para restabelecer função mastigatória e estética, porém, seus efeitos variam conforme o tipo de prótese, as condições de saúde, as expectativas individuais e o acesso aos serviços odontológicos. Evidências indicam associação entre a reabilitação protética e melhora da Qualidade de Vida Relacionada à Saúde Bucal (QVRSB), sobretudo nos domínios funcionais e psicológicos, reforçando a necessidade de abordagens que considerem dimensões mais amplas do cuidado em saúde bucal no envelhecimento^5–10^.

A evolução histórica do uso de próteses dentárias entre pessoas idosas brasileiras, bem como indícios das necessidades de reabilitação protética, pode ser compreendida com base nos levantamentos da Pesquisa Nacional de Saúde Bucal (SB Brasil). Entre 2003 e 2010, observou-se aumento na utilização de próteses totais superiores (57,91 para 63,1%) e inferiores (24,84 para 37,5%), fenômeno de natureza multifatorial relacionado tanto à persistência das perdas dentárias, avaliadas pelo componente "perdido" do CPO-D, quanto à ampliação da oferta e do acesso aos serviços de reabilitação protética. Embora tenha ocorrido redução progressiva do edentulismo, a elevada proporção de dentes perdidos mantém a reabilitação protética removível como a principal estratégia de restabelecimento funcional no contexto dos serviços públicos, uma vez que alternativas como próteses fixas e reabilitações implantossuportadas apresentam oferta limitada no Sistema Único de Saúde (SUS), restringindo seu alcance populacional^9–12^.

Paralelamente, observou-se discreto aumento no uso de reabilitações fixas unitárias. Em 2003, o uso de ponte fixa foi de 0,79% na arcada inferior e de 1,87% na superior, alcançando 3,8% em 2010. Embora ainda pouco frequentes, esses dados sugerem mudanças graduais no perfil das perdas dentárias e no acesso a tratamentos mais conservadores^11–13^, o que reforça a necessidade de investigar a associação entre a condição protética e a QVRSB, cuja investigação exige instrumentos específicos e validados, como o *Oral Impacts on Daily Performance* (OIDP) – Impactos Bucais no Desempenho Diário, em português.

O OIDP avalia os impactos das condições de saúde bucal nas atividades cotidianas, abrangendo dimensões físicas, psicológicas e sociais. No Brasil, sua versão validada apresentou boas propriedades psicométricas em adultos de 50 a 74 anos, sendo adequada para essa população^14^. Estudo recente^15^ também indica que a associação entre a reabilitação protética e a QVRSB não é uniforme: embora a presença de próteses seja um componente importante na avaliação, seus efeitos variam conforme o padrão de perda dentária, por exemplo, se anterior ou com extensão posterior, de modo que alguns tipos de próteses podem não estar associados a melhora significativa desse desfecho.

Apesar desses avanços, persistem lacunas na literatura, sobretudo pela predominância de estudos locais ou com faixas etárias amplas, o que limita análises baseadas em inquéritos populacionais representativos. Ademais, são escassos estudos que avaliem de forma integrada o uso e a necessidade de próteses dentárias e seus impactos funcionais, psicológicos e sociais no contexto do envelhecimento e das desigualdades no acesso aos serviços odontológicos. Assim, permanece limitado o conhecimento epidemiológico nacional que relacione a condição protética ao impacto da saúde bucal nas atividades diárias de pessoas idosas, o que justifica a realização de estudos de abrangência nacional^16,17^.

Desse modo, o presente estudo tem como objetivo verificar a associação entre o uso e a necessidade de próteses dentárias e o impacto da saúde bucal nas atividades diárias de pessoas idosas no Brasil.

## MÉTODOS

### Desenho do estudo e população

Trata-se de um estudo transversal com dados secundários do SB Brasil 2023, que utilizou delineamento amostral estratificado por conglomerados, garantindo representatividade nacional, com cobertura nas cinco macrorregiões, estados e capitais brasileiras^7^. Foram avaliadas 9.745 pessoas idosas com idade entre 65 e 74 anos. Para o presente estudo, indivíduos sem dados clínicos completos foram excluídos, resultando em uma amostra final de 9.696 participantes.

### Coleta de dados e variáveis do estudo

Os dados foram coletados por meio de entrevistas domiciliares e exames clínicos padronizados realizados por examinadores calibrados, seguindo a metodologia da Organização Mundial da Saúde (OMS) para pesquisas em saúde bucal. O questionário coletou informações sociodemográficas (sexo, cor/raça, escolaridade, renda familiar) e relacionadas ao uso de serviços (como utilização de serviços odontológicos e autopercepção sobre o uso e a necessidade de próteses dentárias).

A variável dependente foi a QVRSB, avaliada pelo OIDP, que contempla três domínios: o domínio físico, relacionado a dificuldades em realizar atividades como mastigar ou falar; o domínio psicológico, que abrange impactos sobre bem-estar emocional, autoestima e autopercepção; e o domínio social, referente a limitações nas interações e participação social^18,19^. Conforme metodologia prévia^20^, participantes que relataram ao menos um impacto negativo foram classificados com impacto, enquanto aqueles que responderam negativamente a todos os itens foram considerados sem impacto. O OIDP avalia dificuldades ou impedimentos no desempenho de atividades diárias atribuídos a problemas bucais em um período de referência, constituindo uma medida sintética, validada e amplamente utilizada em estudos epidemiológicos populacionais^21^.

As variáveis independentes foram selecionadas com base em evidências prévias do SB Brasil e em sua relevância teórica para a análise da QVRSB. Foram incluídas variáveis sociodemográficas: sexo (feminino; masculino), cor/raça autodeclarada (branca; preta; parda; amarela; indígena) conforme a classificação do Instituto Brasileiro de Geografia e Estatística (IBGE), escolaridade (sem escolaridade; até 8 anos de estudo; mais de 8 anos de estudo) e renda familiar mensal (<1 salário mínimo; 1–2 salários mínimos; >2 salários mínimos). Também foram consideradas a região de residência (Norte; Nordeste; Centro-Oeste; Sudeste; Sul) e variáveis relacionadas ao uso de serviços odontológicos: utilização de serviços odontológicos no último ano (sim; não) e tipo de serviço utilizado (público; privado; convênio/plano de saúde)^7^.

Quanto às condições clínicas e de reabilitação, analisaram-se: edentulismo (sim; não), uso de prótese dentária (sim; não), necessidade de prótese superior e inferior (sim; não), autopercepção da necessidade de usar ou substituir próteses (sim; não) e presença de próteses ou dentes com implantes (sim; não)^7^. Todas as variáveis foram categorizadas conforme os critérios do SB Brasil, assegurando comparabilidade com estudos nacionais anteriores.

Dados clínicos ausentes (uso e necessidade de prótese dentária e edentulismo; n=25) foram excluídos da amostra, e perguntas que os participantes não souberam ou não quiseram responder foram consideradas como *missing* nas análises.

### Análise estatística

As análises estatísticas foram realizadas por meio do *Statistical Package for the Social Sciences* (SPSS), versão 27.0 (IBM Corp., Armonk, NY, USA). Inicialmente, foram conduzidas análises descritivas com cálculo de frequências absolutas e relativas. As associações entre a QVRSB e as variáveis independentes foram avaliadas por meio de regressão de Poisson com estimador de variância robusta, considerando o desenho amostral complexo do SB Brasil (pesos, estratos e unidades primárias de amostragem).

Na etapa de análises bivariadas, estimaram-se Razões de Prevalência (RP) brutas e respectivos Intervalos de Confiança de 95% (IC95%). Variáveis com p<0,20 foram incluídas no modelo multivariado, permanecendo no modelo final aquelas com p<0,05.

### Considerações éticas

O projeto SB Brasil seguiu os princípios da Declaração de Helsinque e foi aprovado pelo Comitê Nacional de Ética em Pesquisa do Brasil (número de aprovação 4.823.054). Todos os participantes forneceram consentimento informado antes da participação. Como este estudo utilizou dados secundários públicos e anonimizados, nenhuma aprovação ética adicional foi necessária.

#### Declaração de disponibilidade de dados:

Todo o conjunto de dados que dá suporte aos resultados deste estudo está disponível mediante solicitação ao Ministério da Saúde.

## RESULTADOS

A distribuição dos participantes segundo as macrorregiões brasileiras evidenciou maior concentração no Nordeste (33,6%; IC95% 32,6–34,5) e a menor na região Centro-Oeste (13,8%; IC95% 13,1–14,5). A maioria dos participantes era do sexo feminino (61,9%; IC95% 60,9–62,8) e se autodeclarava parda (46,5%; IC95% 45,5–47,5). A média de anos de escolaridade formal foi de 6,96 (±4,57), com apenas 8,2% (IC95% 7,6–8,7) da amostra relatando ter concluído o ensino superior. Em relação à renda, a maioria dos participantes recebia entre 1 e 2 salários mínimos (35,6%; IC95% 34,5–36,7). Uma proporção menor relatou receber mais de 10 salários mínimos (2,1%; IC95% 1,8–2,4) e mais de 5 a 10 salários mínimos (6,2%; IC95% 5,6–6,7) (Tabela 1).

**Tabela 1:** Características sociodemográficas em pessoas idosas brasileiras, SB Brasil 2023.

Variável		n	%	IC95%
Região				
	Norte	2.231	23,0	22,1–23,8
	Nordeste	3.258	33,6	32,6–34,5
	Centro-Oeste	1.344	13,9	13,1–14,5
	Sudeste	1.451	15,0	14,2–15,6
	Sul	1.412	14,6	13,8–15,2
Sexo				
	Masculino	3.693	38,1	37,1–39,0
	Feminino	6.003	61,9	60,9–62,8
Cor/raça autodeclarada				
	Branca	3.578	37,5	36,4–38,4
	Preta	1.350	14,1	13,4–14,8
	Amarela	129	1,4	1,35–1,60
	Parda	4.447	46,5	45,5–47,5
	Indígena	50	0,5	0,4–0,69
Renda familiar (salário mínimo)				
	Até um	1.987	28,3	27,2–29,3
	De 1 a 2	2.501	35,6	34,5–36,7
	Mais de 2	2.531	36,1	34,9–37,1
Anos de estudo				
	Não estudou	1.233	13,0	12,3–13,7
	Até 8	4.573	48,2	47,2–49,2
	Mais de 8	3.675	38,8	37,7–39,7

IC: intervalo de confiança.

Nos últimos anos, 56,2% (IC 95%: 55,2-57,1) dos participantes relataram não ter procurado atendimento odontológico. Entre as 4.206 pessoas idosas que o fizeram (43,8%; IC95% 42,8–44,8), 47,9% (IC95% 46,4–49,4) utilizaram serviços privados, 45,4% (IC95% 43,9–46,9) procuraram serviços públicos e 6,6% (IC95% 5,9–7,4) utilizaram planos de saúde ou seguros. Menos de 1% (0,6%; IC95% 0,4–0,8) relatou buscar outros tipos de serviços. Pouco mais da metade dos participantes (53,2%; IC95% 52,1–54,1) relatou necessidade de uso ou substituição de uma prótese total, e 19,3% (IC95% 18,5–20,1) relataram ter dentes ou próteses apoiados por implantes.

A Figura 1 mostra a prevalência de edentulismo e o uso e a necessidade de próteses dentárias entre pessoas idosas brasileiras. No geral, 35,9% (IC95% 34,9–36,8) dos participantes eram edêntulos, e 70,2% (IC95% 69,3–71,1) relataram usar prótese dentária superior, enquanto 46,7% (IC95% 45,7–47,6) usavam prótese dentária inferior. A necessidade de próteses superiores e inferiores foi relatada por 63,2% (IC95% 62,1–64,1) e 72,1% (IC95% 71,1–72,9) dos participantes, respectivamente. Ao avaliar a necessidade global em ambas as arcadas, 23,8% (IC95% 22,9–24,6) das pessoas idosas não necessitavam de prótese, 22,6% (IC95% 21,7–23,4) necessitavam de próteses parciais em ambas as arcadas; e 23% (IC95% 22,1–23,8), de próteses totais tanto na arcada superior quanto na inferior.

**Figura 1:** Edentulismo, uso e necessidade de próteses dentárias em pessoas idosas brasileiras, SB Brasil, 2023.

Quanto aos tipos utilizados, entre aqueles que relataram usar prótese dentária superior, quase metade (49,8%; IC95% 48,8–50,8) usava prótese total removível; 13,6% (IC95% 12,9–14,2), prótese parcial removível; e 3% (IC95% 2,6–3,3), uma ou mais pontes fixas. Adicionalmente, 2,6% (IC95% 2,3–2,9) usavam próteses totais fixas (*overdentures*), e 1,2% (IC95% 0,9–1,4) utilizavam uma combinação de tipos fixos e removíveis. Para a arcada inferior, 26,2% (IC95% 25,3–27,0) usavam prótese total removível; 14,7% (IC95% 14,0–15,4), prótese parcial removível; e 1,9% (IC95% 1,6–2,2), uma ou mais pontes fixas. Uma pequena proporção (2,6%; IC95% 2,3–2,9) relatou uso de próteses totais fixas e 1,3% (IC95% 1,0–1,5), uma combinação de tipos fixos e removíveis. Ao avaliar as necessidades protéticas, 36,9% (IC95% 35,9–37,8) dos participantes necessitavam de prótese total superior, enquanto 18,2% (IC95% 17,4–19,0) demandavam prótese para repor mais de um dente ausente, e 5,1% (IC95% 4,7–5,6) necessitavam de combinação de próteses fixas e removíveis. Na arcada inferior, 27,3% (IC95% 26,3–28,1) precisavam de prótese total, 32,6% (IC95% 31,6–33,5) necessitavam de reposição para mais de um dente e 9,3% (IC95% 8,7–9,8), de uma combinação de tipos protéticos.

Mais da metade das pessoas idosas (56,1%; IC95% 55,1–57,0) relatou experienciar pelo menos um impacto nas atividades diárias decorrente da saúde bucal, enquanto 2,3% relataram impacto em todas as dimensões avaliadas. Alimentar-se (21,0%) e sorrir (16,1%) foram as atividades mais frequentemente afetadas. Impactos no estado emocional foram observados em 13,3% (Figura 2). As associações entre os impactos bucais e as variáveis independentes são apresentadas na Tabela 2.

**Figura 2:** Impactos bucais no desempenho diário em pessoas idosas brasileiras, SB Brasil, 2023.

**Tabela 2:** Associação entre impactos bucais na qualidade de vida e características sociodemográficas, clínicas e uso e necessidade de próteses em pessoas idosas brasileiras, SB Brasil, 2023.

Variável		Sem impacto	Com impacto	RP	IC95%	p-valor*
		n (%)				
Região do Brasil						
	Centro-Oeste	489 (36,6)	848 (63,4)	1.08	1.02–1.14	**0.005**
	Sul	657 (46,6)	752 (53,4)	0.90	0.85–0.96	**0.002**
	Sudeste	713 (49,5)	727 (50,5)	0.86	0.80–0.91	**<0.001**
	Nordeste	1.459 (45,0)	1.782 (55,0)	0.93	0.89–0.98	**0.006**
	Norte	916 (41,3)	1.303 (58,7)	1		
Sexo						
	Feminino	2.562 (42,9)	3.414 (57,1)	1.04	1.01–1.08	**0.010**
	Masculino	1.672 (45,6)	1.998 (54,4)	1		
Cor da pele (autodeclarada)						
	Indígena	18 (36,0)	32 (64,0)	1.19	0.96–1.47	0.098
	Parda	1.928 (43,6)	2.495 (56,4)	1.05	1.01–1.09	**0.013**
	Amarela	56 (43,8)	72 (56,2)	1.04	0.89–1.22	0.545
	Preta	533 (39,6)	814 (60,4)	1.12	1.06–1.18	**<0.001**
	Branca	1.653 (46,4)	1.910 (53,6)	1		
Escolaridade (anos)						
	Sem escolaridade	482 (39,3)	745 (60,7)	1.13	1.07–1.19	**<0.001**
	Até 8	1.959 (43,0)	2.601 (57,0)	1.06	1.02–1.10	**0.001**
	Mais de 8	1.704 (46,5)	1.960 (53,5)	1		
Renda familiar (salário mínimo)						
	<1	838 (42,3)	1.141 (57,7)	1.08	1.03–1.14	**0.002**
	1–2	1.012 (40,6)	1.481 (59,4)	1.11	1.06–1.17	**<0.001**
	>2	1.183 (46,9)	1.340 (53,1)	1		
Procurou serviço odontológico (último ano)						
	Sim	1.757 (41,9)	2.440 (58,1)	1.06	1.02–1.10	**<0.001**
	Não	2.435 (45,4)	2.934 (54,6)	1		
Tipo de serviço odontológico						
	Público	707 (37,4)	1.183 (62,6)	1.35	1.18–1.54	**<0.001**
	Privado	889 (44,7)	1.101 (55,3)	1.19	1.04–1.36	**0.009**
	Plano de saúde/Convênio	147 (53,6)	127 (46,4)	1		
Autopercepção quanto a uso ou troca da prótese dentária						
	Sim	1.706 (34,1)	3.295 (65,9)	1.46	1.40–1.51	**<0.001**
	Não	2.420 (54,9)	1.986 (45,1)	1		
Dentes/próteses com implantes						
	Sim	855 (46,2)	997 (53,8)	0.94	0.90–0.99	**0.029**
	Não	3.346 (43,3)	4.381 (56,7)	1		
Edentulismo						
	Não	2.620 (42,3)	3.567 (57,7)	1.08	1.04–1.12	**<0.001**
	Sim	1.614 (46,7)	1.845 (53,3)	1		
Uso de prótese superior						
	Não	1.257 (43,8)	1.611 (56,2)	1.02	0.96–1.04	0.933
	Sim	2.977 (43,9)	3.801 (56,1)	1		
Necessidade de prótese superior						
	Não	1.890 (53,1)	1.669 (46,9)	0.76	0.73–0.79	**<0.001**
	Sim	2.344 (38,5)	3.743 (61,5)	1		
Uso de prótese inferior						
	Não	2.128 (41,4)	3.006 (58,6)	1.09	1.05–1.13	**<0.001**
	Sim	2.106 (46,7)	2.406 (53,3)	1		
Necessidade de prótese inferior						
	Não	1.509 (55,9)	1.190 (44,1)	0.72	0.69–0.76	**<0.001**
	Sim	2.725 (39,2)	4.222 (60,8)	1		
Necessidade de prótese superior e inferior						
	Sim	2.917 (39,7)	4.430 (60,3)	1.41	1.34–1.48	**<0.001**
	Não	1.317 (57,3)	982 (42,7)	1		

RP: Razões de Prevalência; IC: intervalo de confiança.

* Associação significativamente estatística (p<0,05).

Houve associação estatística nos números destacados em negrito.

Na análise bivariada (Tabela 2), maior prevalência de impactos bucais foi observada entre participantes da região Centro-Oeste (p=0,005), quando comparados a região Norte (categoria de referência), mulheres (p=0,010), indivíduos pretos (p<0,001) e pardos (p=0,013), aqueles com menor escolaridade (p<0,001) e menor renda familiar (p=0,002), bem como entre os que utilizaram serviços odontológicos públicos (p<0,001). Pessoas idosas que percebiam a necessidade de usar ou substituir prótese total (p<0,001), com edentulismo (p<0,001) e que apresentavam necessidade de próteses dentárias superiores (p<0,001) ou inferiores (p<0,001) também relataram maior prevalência de impactos bucais. Em contraste, indivíduos com dentes ou próteses apoiados em implantes apresentaram menor prevalência de impactos (p=0,029).

No modelo de regressão de Poisson (Tabela 3), o sexo feminino permaneceu significativamente associado a maior prevalência de impactos bucais nas atividades diárias (RP 1,07; IC95% 1,01–1,14; p=0,022). A autopercepção quanto à necessidade de usar ou substituir uma prótese total (RP 1,32; IC95% 1,24–1,41; p<0,001) e a necessidade de prótese dentária superior (RP 1,18; IC95% 1,10–1,27; p<0,001) também se associaram a maior prevalência desses impactos. Por outro lado, ter dentes ou próteses apoiados em implantes associou-se a menor prevalência de impactos bucais nas atividades diárias (RP 0,90; IC95% 0,83–0,97; p=0,006).

**Tabela 3:** Modelo de regressão de Poisson com estimador robusto entre impactos orais no desempenho diário e características sociodemográficas, clínicas e uso e necessidade de próteses em pessoas idosas brasileiras, SB Brasil, 2023.

Variáveis		*B*	RP	IC95%	p-valor
Sexo					
	Feminino	0.073	1.07	1.01–1.14	**0.022**
	Masculino			*Ref.*	
Autopercepção quanto a uso ou troca da prótese dentária					
	Sim	0.284	1.32	1.24–1.41	**<0.001**
	Não			*Ref.*	
Dentes/próteses com implantes					
	Sim	-0.104	0.90	0.83–0.97	**0.006**
	Não			*Ref.*	
Necessidade de prótese superior					
	Sim	0.172	1.18	1.10–1.27	**<0.001**
	Não			*Ref.*	

RP: Razões de Prevalência; IC: intervalo de confiança.

*Ref.:* categoria de referência da análise.

Houve associação estatística nos números destacados em negrito.

## DISCUSSÃO

Os achados deste estudo evidenciam que a saúde bucal continua exercendo impacto significativo nas atividades diárias das pessoas idosas brasileiras. Mais da metade dos participantes relatou pelo menos um impacto bucal, sendo alimentação, sorriso e aspectos emocionais os domínios mais afetados. Observou-se maior prevalência de impacto entre mulheres, indivíduos com autopercepção da necessidade de usar ou substituir próteses dentárias e aqueles com necessidade de prótese superior, enquanto a presença de dentes ou próteses apoiados em implantes mostrou-se fator protetor. Além das condições clínicas objetivas, fatores subjetivos e sociodemográficos desempenham papel central na determinação dos impactos bucais sobre a vida cotidiana, reforçando a complexidade da relação entre perdas dentárias, reabilitação protética e a QVRSB na população idosa brasileira.

O edentulismo, embora não seja o foco central deste estudo, permanece um importante marcador das condições de saúde bucal das pessoas idosas brasileiras e fornece contexto para a compreensão da elevada necessidade de reabilitação protética observada. Estudos prévios^13,22–24^ do SB Brasil documentam um declínio progressivo das taxas de edentulismo ao longo das décadas; contudo, a magnitude ainda registrada reflete um histórico de práticas odontológicas mutiladoras e de acesso limitado a ações preventivas e tratamentos conservadores^25^. A predominância de próteses totais removíveis, descrita na literatura nacional, especialmente nos levantamentos do SB Brasil e em estudos sobre a produção de próteses no SUS, reforça que a reabilitação protética convencional permanece como a principal estratégia de enfrentamento da perda dentária entre pessoas idosas no contexto dos serviços públicos de saúde no Brasil^6,13^.

Observou-se elevada prevalência de necessidade de prótese superior e inferior, corroborando investigações anteriores realizadas no Brasil^26,27^, especialmente em contextos marcados por desigualdades no acesso aos serviços odontológicos. Apesar da expansão das políticas públicas de saúde bucal, a oferta pública de próteses ainda se mostra insuficiente para atender à demanda existente. Ademais, a perda dentária ocorreu, em grande parte, durante a vida adulta, refletindo práticas odontológicas historicamente centradas em abordagens mutiladoras e em um modelo assistencial predominantemente curativo. Assim, a elevada necessidade de próteses identificada no SB Brasil 2023 expressa demandas atuais do sistema de saúde e o acúmulo de iniquidades e lacunas assistenciais ao longo do curso de vida.

Os dados deste estudo também indicam mudança em relação às edições anteriores do SB Brasil: embora tenha ocorrido redução do edentulismo, a necessidade de reabilitação protética manteve-se elevada, destacando-se a crescente demanda por próteses totais superiores. Esse achado pode ser parcialmente explicado pelo caráter autorreferido dessa informação, uma vez que a ausência, inadequação ou o desgaste das próteses superiores tende a gerar maior percepção de prejuízos estéticos, funcionais e psicossociais, como dificuldades na alimentação, na fala, no sorriso e na interação social^28–30^. Desse modo, a maior demanda por próteses superiores pode refletir tanto condições clínicas objetivas quanto uma ampliação da percepção dos impactos da saúde bucal nas atividades diárias, conforme mensurado pelo OIDP^31,32^.

A discrepância entre o uso e a necessidade de próteses, especialmente na arcada inferior, pode ser explicada por barreiras socioeconômicas e de acesso aos serviços. A maior necessidade protética entre indivíduos de menor renda e escolaridade reflete dificuldades financeiras e limitações no acesso a serviços odontológicos especializados no setor público. Evidências nacionais e internacionais^22,24,33–36^ indicam associação consistente entre condições socioeconômicas desfavoráveis e piores condições de saúde bucal entre pessoas idosas. Soma-se a esse cenário a elevada demanda reprimida nos serviços públicos de atenção especializada, que resulta em longos tempos de espera e postergação dos tratamentos reabilitadores^37^.

Além do acesso, a adequação das próteses utilizadas constitui componente central na compreensão da discrepância entre necessidade e uso. Evidências clínicas demonstram que parcela considerável das próteses utilizadas por pessoas idosas apresenta deficiências de retenção, estabilidade e adaptação funcional, comprometendo a função mastigatória, o conforto e a satisfação com o tratamento, o que aumenta a probabilidade de necessidade de substituição ou abandono do uso^30,32^. Esse cenário contribui para a manutenção de impactos negativos na QVRSB e para o aprofundamento das desigualdades no cuidado.

A autopercepção da saúde bucal mostrou-se fortemente associada à necessidade percebida de uso ou troca de próteses e maior prevalência de impactos nas atividades diárias. Evidências nacionais indicam que uma autopercepção positiva pode estar relacionada à ausência percebida de necessidade de tratamento, mesmo diante de condições clínicas desfavoráveis^38^, sugerindo adaptação às limitações do envelhecimento^39^. Em contrapartida, estudos demonstram que uma autopercepção negativa está associada a maior impacto funcional e psicossocial, maior número de queixas e menor utilização de serviços odontológicos^30,40^, evidenciando a relevância dos fatores subjetivos na experiência da saúde bucal.

A interface entre uso/necessidade de prótese e qualidade de vida, mensurado pelo OIDP, ganha destaque ao se constatar que mais da metade das pessoas idosas relatou ao menos um impacto bucal em suas atividades diárias. Esses achados estão alinhados à literatura internacional^41–43^, que aponta associação consistente entre perda dentária, necessidade de prótese e pior QVRSB. No contexto brasileiro, os impactos mais frequentes sobre alimentação, sorriso e estado emocional reforçam que a demanda por prótese ultrapassa o caráter técnico, envolvendo a autoestima, interações sociais e o bem-estar psicológico^44^.

Observou-se, ainda, que o sexo feminino se associou a maior probabilidade de relatar impactos da saúde bucal nas atividades diárias, resultado consistente com a literatura que aponta diferenças de gênero na percepção de impactos sociais e psicológicos relacionados à saúde bucal^45,46^. A persistência do sexo feminino como determinante significativo do OIDP indica a necessidade de estratégias de atenção odontológica sensíveis ao gênero, voltadas à redução dessas desigualdades.

Os condicionantes estruturais para a oferta e o uso de próteses também merecem destaque. A amostra evidencia que parcela expressiva dos atendimentos odontológicos ocorreu tanto em serviços privados quanto públicos, indicando que a rede pública ainda responde por parte substancial da demanda protética. Esse cenário, aliado ao perfil socioeconômico menos favorecido, aponta para a persistência de desigualdades na oferta e no acesso aos serviços. Estudo brasileiro destaca que regiões com menor Índice de Desenvolvimento Humano (IDH) e menor densidade de infraestrutura odontológica apresentam maior necessidade de prótese e menor uso^30^, reforçando a urgência de políticas voltadas à reabilitação protética no âmbito do SUS, com ênfase em equidade.

No contexto do SUS, a reabilitação protética deveria ser predominantemente ofertada na Atenção Primária à Saúde (APS), por sua capilaridade, acessibilidade e longitudinalidade do cuidado^47^. Entretanto, estudos prévios^48–50^ revelam que um número ínfimo de equipes de saúde bucal na APS efetivamente oferece serviços de prótese dentária, concentrando esses serviços na atenção especializada ou no setor privado.

Quanto ao tipo de reabilitação, próteses totais removíveis, especialmente quando mal adaptadas, estão associadas a maior impacto negativo na qualidade de vida^51^. Estudos nacionais apontam associação entre uso de próteses parciais inferiores e maior risco de impacto no OIDP^32,52^, reforçando que não basta ampliar a oferta, sendo fundamental garantir qualidade, adequação e acompanhamento contínuo das próteses instaladas^30^. Assim, a elevada prevalência de próteses removíveis totais e o expressivo percentual de necessidade não atendida sugerem que não basta fornecer prótese, mas garantir sua adequação, manutenção e reabilitação completa para minimizar os impactos na qualidade de vida.

O uso de próteses implantossuportadas apresentou efeito protetor na qualidade de vida das pessoas idosas, corroborando estudos que demonstram melhora significativa após a instalação de *overdentures* ^53,54^. Essas reabilitações oferecem maior estabilidade e retenção em comparação às próteses removíveis convencionais, favorecendo melhor mastigação, conforto, fala e satisfação com a aparência^29,30,55^. Contudo, a baixa prevalência desse tipo de reabilitação reflete não somente questões socioeconômicas e de acesso, mas também limitações estruturais do sistema de saúde brasileiro. Embora o SUS financie esses procedimentos desde 2010, sua realização permanece incipiente^56,57^, reforçando a necessidade de políticas que ampliem o acesso a essas tecnologias^58^.

Este estudo apresenta limitações inerentes ao delineamento transversal, que impede inferências causais. Adicionalmente, a avaliação da qualidade de vida baseou-se em autorrelato, sujeita a vieses de memória e subjetividade. Entretanto, a representatividade nacional da amostra, o rigor metodológico do SB Brasil e o uso de instrumentos validados conferem robustez aos achados. A análise multivariada permitiu controlar potenciais confundidores.

Em consonância com a literatura^59^, a mensuração dos impactos por meio do OIDP constitui ferramenta estratégica para a identificação de grupos vulneráveis e para o monitoramento das políticas públicas. Desse modo, os resultados reforçam que a condição protética ocupa papel central na relação entre saúde bucal e qualidade de vida das pessoas idosas no Brasil, evidenciando a necessidade de políticas que ampliem o acesso equitativo, qualificado e contínuo à reabilitação protética no âmbito do SUS, com atenção às desigualdades regionais, socioeconômicas e de gênero.
